# Case of Hæmoptysis

**Published:** 1858-05

**Authors:** Jas. M. Kindall

**Affiliations:** Wabash, Indiana


### ARTICLE VI.

WABASH, INDIANA, MARCH 27, 1858.

N. S. DAVIS, M. D.,—Allow me to offer for publication in the *Chicago Medical Journal*, the following *case*. I think it interesting, from the fact, that no trace of phthisis is known in the history of my ancestry, so far back as grandparents on either side. Could there be a phthisical origin farther back? I know of no cause save two years' confinement in an unventilated studio.

### CASE OF HÆMOPTYSIS.

I, J. M. K., aged twenty-two years, a medical student, of a sanguine bilious temperament, was attacked August 1st, 1857, with hemorrhage from the lungs (while sitting in the office), ejected half a pint of florid blood, having no premonitory symptoms save an immediate ebullition in the chest; had been rather anæmic for several months; had a tooth extracted carefully by a dentist on the 4th of July, 1857; hemorrhage proceeded from the orifice, and was not arrested, notwithstanding the efforts made to do so, for nearly twenty-four hours; pulmonary hemorrhage recurred every night for several successive nights, sometimes twice in the same night; ten grs. of plumb. acid, and ¼

gr. ipecac., had been administered every four hours, together with gallic acid, wine of ergot, acid. tannic., and other astringents; pulse generally 100 to 110 per minute when from under the influence of sedatives, but was easily controlled by the use of tinc. of verat. viride. In addition to the above, rem. quinine was administered, to the effects of which, as well as to astringents, the disease was in no way amenable, but continued to recur at least once in forty-eight hours, up to September 2d, a space of thirty-two days. During the last few days of the disease, terebinthina was administered in fifteen to twenty drop doses *every three hours*, to the effects of it, and *it alone*, the disease yielded. I continued the use of trebin., in small doses, for a week or two after the hemorrhage ceased. I had a slight cough during the time the hemorrhage lasted, but have coughed little since; expectorate some, the appearance of which is not characteristic of pus as described in medical works. It is now five months since the last hemorrhage, and I feel quite well, but am using oleum jec. acelli in rye whisky, as an agent to ward off phthisis, for I think I have had a premonitory symptom at least of its incipient stage. I have not resumed the study or practice yet; think I had best spend the summer on some of the northern lakes, and think I will be an inmate of the Rush Medical College next fall and winter; would have been last, but was prevented by ill health.—Yours truly, a lover of Esculapian science, JAS. M. KINDALL.

